# Ask me in your own words: paraphrasing for multitask question answering

**DOI:** 10.7717/peerj-cs.759

**Published:** 2021-10-27

**Authors:** G. Thomas Hudson, Noura Al Moubayed

**Affiliations:** Department of Computer Science, Durham University, Durham, United Kingdom

**Keywords:** Question answering, Paraphrasing, Multitask learning, Dataset

## Abstract

Multitask learning has led to significant advances in Natural Language Processing, including the decaNLP benchmark where question answering is used to frame 10 natural language understanding tasks in a single model. In this work we show how models trained to solve decaNLP fail with simple paraphrasing of the question. We contribute a crowd-sourced *corpus* of paraphrased questions (PQ-decaNLP), annotated with paraphrase phenomena. This enables analysis of how transformations such as swapping the class labels and changing the sentence modality lead to a large performance degradation. Training both MQAN and the newer T5 model using PQ-decaNLP improves their robustness and for some tasks improves the performance on the original questions, demonstrating the benefits of a model which is more robust to paraphrasing. Additionally, we explore how paraphrasing knowledge is transferred between tasks, with the aim of exploiting the multitask property to improve the robustness of the models. We explore the addition of paraphrase detection and paraphrase generation tasks, and find that while both models are able to learn these new tasks, knowledge about paraphrasing does not transfer to other decaNLP tasks.

## Introduction

Recent progress in Natural Language Processing (NLP) has led to improved performance across a wide range of language understanding problems (www.gluebenchmark.com/leaderboard). A key component of these advances is the use of knowledge transferred from other tasks, most prominently from language modelling (Peters et al., 2018; Howard & Ruder, 2018; Devlin et al., 2019).

**Figure 1:**
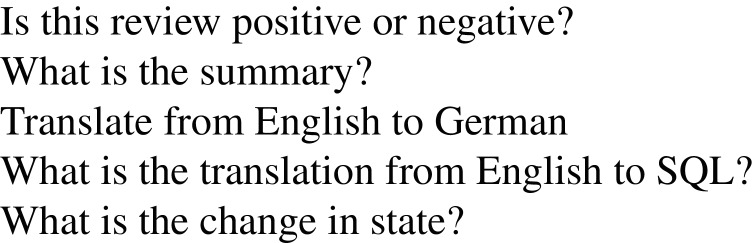
Examples of fixed questions for decaNLP.

McCann et al. (2018) developed a new NLP benchmark: the Natural Language Decathlon (decaNLP). This challenges a single model to perform 10 Natural Language Understanding (NLU) tasks by framing each task as question answering (Fig. 1). For example, when solving a translation task, a model is asked the question “Translate from English to German”, given a paragraph of English text as the context, and is expected to output the translation of the context in German as the answer. The key appeal of this task design is that it favours models where all parameters are shared between all tasks and adding new tasks only requires additional training data, not redesigning the model. As well as decaNLP, McCann et al. (2018) proposed the Multitask Question Answering Network (MQAN) as a neural network architecture for solving the 10 decaNLP tasks. More recently, models such at T5 (Raffel et al., 2020) have explored a similar text-to-text paradigm.

It’s important to note that these questions are not intended to be simple wake-up words (WUWs), but as fully grammatical sentences. A major motivation for decaNLP is the ability to support zero-shot learning. By forming a new task as question answering, MQAN can solve tasks which are variations of the ones it was trained on-For example, MQAN trained to detect sentiment using the question “Is the review positive or negative?” should also be able to answer similar questions for related tasks such as “Is the sentence happy or is it angry?”. This requires models to understand the meaning contained within the questions rather than just treating them as simple WUWs. We suggest that robustness to paraphrasing is a key first step (necessary but not sufficient) in making the idea of more general zero-shot learning feasible within the decaNLP paradigm.

We also envisage real-world applications where a user can interact with a system by asking questions about some context document in order to solve a task. Outside a controlled academic context where we control the prompt, a user should to be able to express the task they want the system to solve in a natural way. We could imagine this being part of a voice assistant, where a user asking “Translate this for me”, or “What’s the translation?” are both equally valid.

In this work, we systematically analyse the robustness of text-to-text models to paraphrasing, as well as explore techniques to improve it *via* adding paraphrase detection and paraphrase generation as additional tasks. For this purpose, we contribute a crowd-sourced *corpus* (www.github.com/ghomasHudson/paraphraseDecanlpCorpus) of paraphrased questions: PQ-decaNLP.

More importantly, we annotate the PQ-decaNLP *corpus* using a paraphrase typology, allowing new analysis of the specific types of paraphrase phenomena which cause the model to fail. We find that the performance is significantly harmed by simple transformations such as exchanging the order of words and changing the sentence modality from questions to imperative commands, but that training using PQ-decaNLP questions greatly improves the robustness to paraphrasing.

## Related work

In this section we provide a general overview of multitask learning, the unique setting provided by decaNLP, as well as a description of paraphrasing from a natural language processing perspective.

**Paraphrasing:** Paraphrasing is often defined as ‘sameness of meaning’ (Vila, Martí & Rodríguez, 2014). This however is ambiguous as there are many degrees of ‘sameness’, and the boundary between paraphrasing and other phenomena (*e.g*. co-reference, inference) is often unclear.

In NLP, paraphrasing is generally studied from a machine learning perspective, with notable interest surrounding paraphrase identification for plagiarism detection (El Desouki & Gomaa, 2019; Hunt et al., 2019; Altheneyan & Menai, 2020). Recent advances in language models have shown state-of-the-art performance on this task and the related task Natural Language Inference (Yang et al., 2019; Devlin et al., 2019). The standard *corpus* used for evaluation is the Microsoft Research Paraphrase *Corpus* (Dolan & Brockett, 2005), which consists of annotated pairs extracted from news articles. Quora Question Pairs (QQP) (www.quora.com/q/quoradata/First-Quora-Dataset-Release-Question-Pairs) is a larger dataset, formed of questions submitted to the website Quora, and is often used for training models (Imtiaz et al., 2020; Tomar et al., 2017; Li et al., 2019).

Additionally, various methods have been developed to make NLP models more robust to paraphrasing their input (Ribeiro, Singh & Guestrin, 2018; Minervini & Riedel, 2018; Iyyer et al., 2018). Many of these methods consist of automatically generating variations of the input, feeding each into the model, then ensembling the answers. Dong et al. (2017) perform this *via* back-translation, while Buck et al. (2018) explore an approach based on an agent which has been trained using reinforcement learning to reformulate the input to maximise the performance of the final model. To enable zero-shot learning on decaNLP, the model should be robust to more complex types of paraphrasing, particularly at the semantics level.

To better categorise paraphrase phenomena, typologies can be constructed based on the understanding of paraphrasing from different fields, primarily theoretical linguistics, discourse analysis, and computational linguistics.

In computational linguistics, typologies are often formed as lists of specific paraphrase mechanisms, grouped into general classes for use in a particular application. Defined at such a low level, these are incomplete descriptions of paraphrasing and cannot be easily transferred to other languages. Vila Rigat et al. (2011) developed a typology specifically with NLP applications in mind. Their hierarchical approach has been used to tag plagiarism corpora (Barrón-Cedeño et al., 2013), and the influential Microsoft Research Paraphrase *Corpus* (MSRPC-A) (Vila et al., 2015). The typology consists of 20 paraphrase types and is hierarchical, where paraphrase types are grouped by the level that the change occurs (*e.g*. morphological, lexical, semantics). Commonly occurring types include Addition/deletion (adding or removing lexical/functional units), Same-polarity substitution (changing one lexical/functional unit with another of the same meaning), Sentence modality changes (Changing the modality of a sentence, *e.g*. from an imperative command to a question), Synthetic/analytic substitutions (swapping a synthetic for an analytic structure *e.g*., “smarter than everyone else” to “the smartest”), order (swapping the order of some sentence element, *e.g*. the order of items in a list).

**Multitask learning:** Traditionally, machine learning models are trained to perform well on a single task in isolation. This differs greatly from how humans learn new tasks-by relying on prior experience solving related problems. Multitask learning seeks to emulate this process by training models to solve multiple objectives simultaneously.

Methods for multitask learning can be divided into three main schemes: soft parameter sharing, hierarchical sharing, and hard parameter sharing. In soft-parameter sharing, each task uses a subset of the parameters, but these are constrained using regularisation techniques (such as *l*_2_ or trace norm) to favour similar values (Duong et al., 2015; Yang & Hospedales, 2017). Hierarchical approaches make explicit use of the theorised relationships between tasks, where some tasks (*e.g*. named entity recognition) require simple ‘low-level’ reasoning, and others (*e.g*. relation extraction) build on this to enable deeper, more complex understanding. These relationships are mirrored in hierarchical approaches, where layers close to the input are used to solve the low-level tasks (Sanh, Wolf & Ruder, 2018; Hashimoto et al., 2017). In hard parameter sharing, a proportion of the parameters are shared between all tasks and the remainder are task-specific-commonly the output layers (Caruana, 1993). This is a stronger form of multitask learning with whole layers used by multiple tasks.

In this work we focus on decaNLP (McCann et al., 2018) which is a strong form of hard-sharing.

### DecaNLP

The decaNLP challenge (McCann et al., 2018) frames multiple tasks as question answering, and is an extreme case of hard parameter sharing where all the parameters are shared (without any task-specific parameters). This approach has key advantages, primarily that new tasks can be added without any modification to the model architecture, only requiring changes to the dataset to frame the task as a question. Table 1 shows the 10 tasks included in decaNLP. Each one uses standard, publicly-available datasets and metrics. These metrics are simply aggregated to give an overall ‘decaScore’.

**Table 1:** Tasks included in decaNLP with example questions, contexts, and answers. EM = Exact Match, nF1 = Normalised F1, cF1 = *corpus*-level F1, dsEM = dialogue state EM, lfEM = logical form EM.

Task (Dataset)	Metrics	Question	Context	Answer
Translation (IWSLT)	BLEU	What is the translation from English to Geman?	Most of the planet is ocean water.	Der Großteil der Erde ist Meerwasser.
Summarization (CNN/DM)	ROUGE	What is the summary?	Harry Potter star Daniel Radcliffe gains access to a reported £320 million fortune…	Harry Potter star Daniel Radcliffe gets £320M fortune…
NLI (MNLI)	EM	Hypothesis: Some men are playing a sport.	A soccer game with multiple males playing.	Entailment
Sentiment Analysis (SST)	EM	Is this sentence positive or negative?	The product was a pleasure to use.	Positive
Semantic Role Labelling (QA-SRL)	nF1	What is equipped to do something?	Ballast tanks are equipped to change a ship’s trim.	Ballast tanks
Relation Extraction (QA-ZRE)	cF1	Who was in charge of Kraków?	The current President of Kraków, is Jacek Majchrowski.	Jacek Majchrowski
Dialogue State Tracking (WOZ)	dsEM	What is the change in dialogue state?	Are there any French restaurants?	food: French
Semantic Parsing (WikiSQL)	lfEM	What is the translation from English to SQL?	The table has column names… Who is the player that wears number 42?	SELECT Player FROM table WHERE No. = 42
Commonsense Reasoning (MWSC)	EM	Who feared violence? councilmen or demonstrators?	The city councilmen refused the demonstrators a permit because they feared violence.	councilmen
Question Answering (SQuAD)	nF1	What causes precipitation to fall?	In meteorology, precipitation is any product of condensation that falls under gravity…	gravity

**Figure 2:**
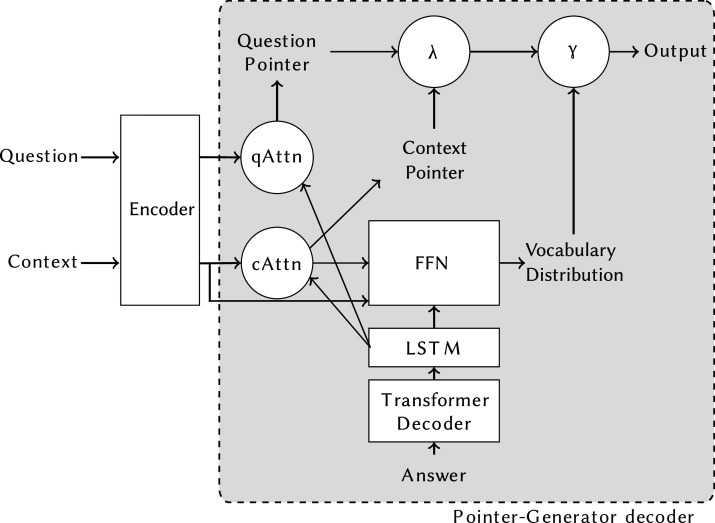
MQAN model overview. It is structured as an encoder-decoder model, with a pointer-generator decoder chooses between copying from the question, context, or an external vocabulary without explicit supervision.

Figure 2 shows the model proposed by McCann et al. (2018) to solve decaNLP: the MQAN (Multitask Question-Answering Network). This network is based on encoder-decoder models for abstractive question answering, notably employing a pointer-generator mechanism for creating the output, a technique commonly applied to summarisation (See, Liu & Manning, 2017). MQAN generalised the pointer mechanism to allow it to construct the output from the question, context or an external vocabulary. This is a modification particularly important for decaNLP tasks where the question may contain the class labels (*e.g*. “Is this review *positive*, or *negative*?”), the context can contain key phrases (as in summarisation), or words can only be in the vocabulary (as in translation). The encoder of the model uses a BiLSTM encoder, dual co-attention, and self-attention to encode the question and context sequences ensuring that long-term dependencies are captured and information is shared between the sequences. The full details of this model can be found in McCann et al. (2018).

Raffel et al. (2020) build on decaNLP to explore a similar text-to-text paradigm using a transformer model trained using simple keyword prompts (*e.g*. “summarize:”, “cola sentence:”). This model (named T5) was constructed after a series of experiments comparing different architectures, unsupervised objectives and multitask-learning strategies. The final model organizes its transformer blocks in an encoder-decoder structure, pretraining it using a BERT-style denoising objective. This model achieved state-of-the-art performance on 18 NLP tasks.

## Methodology

We present our methodology for the two parts of our work: 1. Our new PQ-decaNLP dataset which we use to analyse how the existing models perform when provided with paraphrased questions, and 2. Proposed improvements to the model training to increase the performance on paraphrased questions.

### The PQ-decaNLP dataset

**Figure 3:**
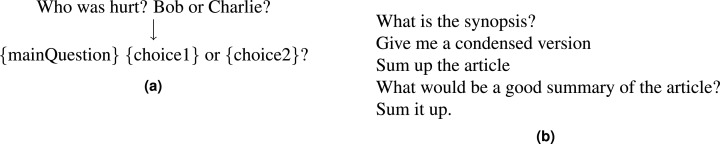
(A) Template transformation for the Modified Winograd Schema Challenge (MWSC). (B) Examples from the paraphrase corpus for the summarisation task.

We create a paraphrased version of decaNLP questions: PQ-decaNLP, using the crowdsourcing platform Amazon Mechanical Turk (www.mturk.com). Workers were given a description of a decaNLP task and were asked to provide five paraphrases of the fixed question. For tasks which have instance-specific information, for example NLI, where the hypothesis is embedded in the questions, we transform them into generic templates (Fig. 3A).

Of the 10 decaNLP tasks, we limit our work to the seven tasks which have a fixed question template, removing Question Answering, Semantic Role Labelling, and Zero-Shot Relation Extraction where the question differs for every example. Techniques for improving the robustness of these excluded tasks are not decaNLP-specific and have been widely studied elsewhere (Fader, Zettlemoyer & Etzioni, 2013; Bordes, Chopra & Weston, 2014; Dong et al., 2017).

These were then inspected using the open source project LanguageTool (www.languagetool.org) for spelling and grammar mistakes. Additionally, we removed paraphrases which did not preserve the meaning of the original question, were ungrammatical, or were duplicates, *via* a manual review. We accepted 73.1% of the paraphrases, rejecting 3.7% due to grammatical errors, 2.9% due to duplication, and 20.3% which were not paraphrases of the original.

**Figure 4:**
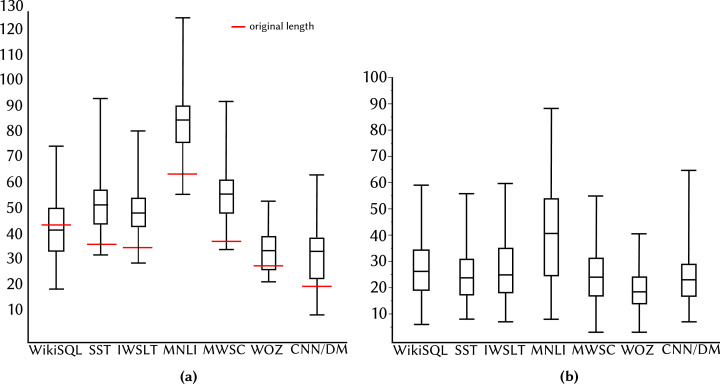
(A–B) Summary of *corpus* statistics.

Figure 3B shows examples of paraphrases for the summarisation task. We collect 100 paraphrases per task to ensure a variety of paraphrases types while minimising duplication. The resulting 700 paraphrases (100 per task) are split 70/30 into train/test sets. Figure 4A shows the distribution of question lengths for the paraphrase *corpus* compared with the original decaNLP questions. We see that the majority of the paraphrases are longer than the original fixed question, suggesting that authors tend to add complexity when paraphrasing, contrary to the findings of Barrón-Cedeño et al. (2013). This may be because the decaNLP questions are already simplistic in comparison to the more complex sentences from Project Gutenberg (www.gutenberg.org) books as used in the work of Barrón-Cedeño et al. (2013). Figure 4B shows edit distances, with paraphrases of MNLI differing most from the original question.

For evaluating the models, we define the PQ-decaScore as the sum of the task-specific metrics for seven tasks that we consider on the PQ-decaNLP dataset, similarly to how the decaScore of McCann et al. (2018) is defined over the full set of 10 tasks.

#### Annotation

**Figure 5:**
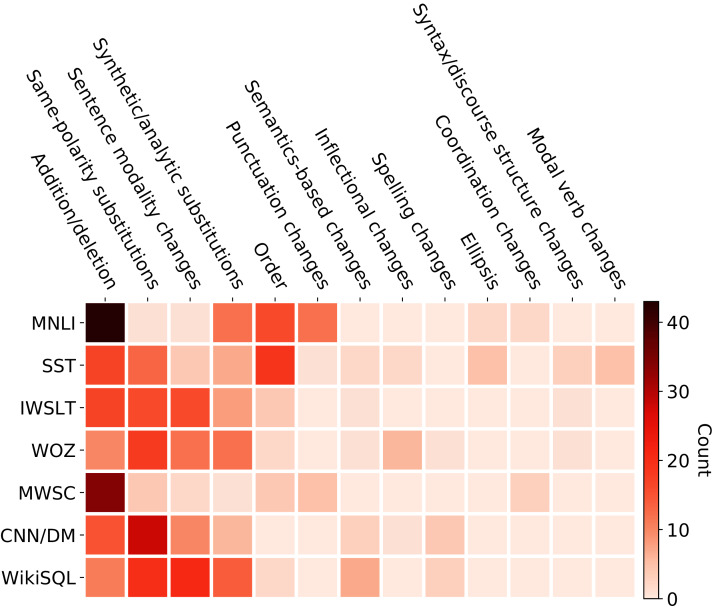
Counts of the paraphrase phenomena occurring in the test set.

To gain an understanding of exactly which kinds of paraphrasing reduce the performance of the models, we hand-annotate the PQ-decaNLP test set using the typology of Vila, Martí & Rodríguez (2014). As our dataset exclusively contains questions and imperative statements, we only observe a subset of the paraphrase phenomena as shown in Fig. 5. Same-polarity and Addition/Deletion are the most frequent phenomena in our dataset, confirming the similar findings of Vila et al. (2015) on the P4P, and MSRP-A datasets. We see no examples of Negation, and Opposite-polarity, but higher frequencies of sentence modality change.

### Proposed improvements

Investigating the performance of the models on the PQ-decaNLP paraphrase questions, we find lower scores across all tasks, indicating the models are not robust to paraphrasing of the question. These results and analysis are presented in the Results and Discussion section.

To enhance the robustness of the models, we propose several improvements. As our focus is the exploration of the existing models, we restrict our scope to modification of the data (adding/modifying decaNLP tasks) rather than the model architectures themselves.

For all our experiments, we use the top-performing version of the Multitask Question Answering Network (MQAN) presented in the original work of McCann et al. (2018). This model is trained on all ten decaNLP tasks using an anti-curriculum strategy, where the model is first trained on the SQuAD dataset alone (phase one) before sampling batches “round-robin”, from all the tasks in a fixed order (phase two). We use the t5-base version of the T5 model, pretrained on the C4 *corpus* and finetuned following the procedure in Raffel et al. (2020).

**Training on PQ-decaNLP:** Our first method is to directly train the model on PQ-decaNLP. For each example in the decaNLP training set, we perform uniformly distributed random selections to pick a question from the PQ-decaNLP training set to replace the fixed question. This directly trains the model to consider different paraphrases of the question.

**Adding paraphrase tasks:** Secondly, we propose to exploit the multitasking abilities of the models by adding a new task to indirectly teach the model about paraphrasing in general. To do this, we introduce a paraphrase detection task (identifying whether two sentences are a paraphrase pair), or a paraphrase generation task (generating a paraphrase of the given sentence).

Introducing a new task rather than changing the data of existing tasks has the advantage of preserving the ease of extending decaNLP to additional tasks in the future. Using this approach, new tasks can still be added with fixed questions as before. We need only a dataset of general paraphrase pairs.

For paraphrase detection we ask the question: “*[paraphraseCandidate1]–paraphrase, or nonparaphrase?*”, and provide *[paraphraseCandidate2]* as the context (where *[paraphraseCandiate1]* and *[paraphraseCandiate2]* are the two sequences in a possible paraphrase pair). We expect that similar to the existing decaNLP tasks of SST and NLI, the model will learn to select the output classes ‘paraphrase’ and ‘nonparaphrase’ from the question.

Paraphrase generation is framed as a sequence-to-sequence task using the question: “*What is the paraphrase?*” and *[paraphraseCandidate1]* as the context. We train the model with the target of *[paraphraseCandidate2]*.

Additionally, we experiment with variants of these tasks which don’t explicitly instruct the model to perform paraphrase detection/generation in the question (The ‘Without task information’ setting). For detection, we use the question: *[paraphraseCandidate1]*, context: *[paraphraseCandidate2]* and train the model to output ‘yes’ or ‘no’ from the external vocabulary. This directly trains the model to paraphrase the entire question. For paraphrase generation, we use *[paraphraseCandidate1]* as the question with a blank context. We train the model to generate *[paraphraseCandidate2]*. These are not valid decaNLP tasks but can be used as pretraining tasks, with the task specific information given by the answer the task is supervised on.

Table 2 shows examples of these formulations with a sample paraphrase pair.

**Table 2:** Example of paraphrase question formulation.

Detection	Generation
**With task information**	
Question: “How do you start a bakery?”– paraphrase, or nonparaphrase?	What is the paraphrase?
Context: “How can one start a bakery business?”	“How do you start a bakery?”
Answer: “paraphrase” or “nonparaphrase”	“How can one start a bakery business?”
**Without task information**	
Question: “How do you start a bakery?”	“How do you start a bakery?”
Context: “How can one start a bakery business?”	
Answer: “yes” or “no”	“How can one start a bakery business?”

## Results and Discussion

To examine the robustness to paraphrasing, we evaluate on the PQ-decaNLP dataset. In Table 3 we observe a decrease in scores across many tasks, with the MQAN model showing the largest decreases in performance for IWSLT, MNLI, CNN/DM, and SST, and the T5 model with the CNN/DM and MWSC tasks.

**Table 3:** Validation metrics for decaNLP and PQ-decaNLP datasets: We show paraphrase detection and generation in settings which indicate the task in the question (with task information), and for those where task information is only indicated by the supervision (without task information). The last model is trained only on PQ-decaNLP questions (PQ-decaNLP trained). We report different metrics for each task as described in Table 1. The decaScore and PQ-decaScore reported here are the sum of the task specific metrics.

Dataset	Dataset	Base	With task information		Without task information		PQ-decaNLP trained
			Detection	Generation	Detection	Generation	
MQAN							
decaNLP	IWSLT	13.7	14.8	14.5	13.0	14.7	15.9
	CNN/DM	24.6	24.5	25.1	24.7	24.0	24.6
	MNLI	69.2	71.3	70.9	69.2	71.3	71.0
	SST	86.4	86.6	84.7	85.1	86.4	86.2
	WOZ	84.1	81.8	84.0	83.6	87.2	84.2
	WikiSQL	58.7	63.3	57.3	60.8	65.2	65.6
	MWSC	48.4	40.2	40.2	51.2	36.6	45.1
	decaScore	385.1	382.5	376.7	387.6	384.4	392.6
PQ-decaNLP	IWSLT	2.8	4.5	3.1	5.7	4.8	14.4
	CNN/DM	8.4	10.8	10.3	11.1	7.5	22.5
	MNLI	43.4	16.1	42.6	20.2	48.3	69.9
	SST	23.4	21.0	72.7	23.2	32.7	84.6
	WOZ	71.2	66.4	62.0	65.3	52.3	78.5
	WikiSQL	55.8	26.8	41.0	28.1	54.9	62.9
	MWSC	40.0	26.5	36.7	19.2	26.4	38.1
	PQ-decaScore	245.0	172.1	268.4	172.8	226.9	370.9
T5							
decaNLP	IWSLT	31.8	30.0	31.6	31.5	31.8	31.0
	CNN/DM	31.9	32.0	32.5	32.0	32.1	32.3
	MNLI	76.8	76.4	76.4	76.7	77.3	75.8
	SST	91.2	90.0	90.8	89.9	89.9	87.2
	WOZ	85.3	83.7	80.8	81.9	82.4	80.8
	WikiSQL	58.9	60.0	58.5	59.5	59.5	56.5
	MWSC	54.9	50.0	50.0	48.8	45.1	48.8
	decaScore	430.8	422.1	420.6	420.3	418.1	412.4
PQ-decaNLP	IWSLT	25.6	24.7	22.3	25.8	21.3	31.3
	CNN/DM	22.7	19.6	21.3	22.5	20.4	25.3
	MNLI	73.3	72.6	72.6	73.3	74.1	75.5
	SST	89.9	89.7	89.8	89.5	89.5	87.9
	WOZ	75.1	79.6	78.9	76.8	70.3	79.1
	WikiSQL	57.9	58.4	57.2	57.9	57.9	56.0
	MWSC	44.2	40.5	35.5	39.6	34.3	48.1
	PQ-decaScore	388.7	385.1	377.6	385.4	367.7	403.2

Trained on the original decaNLP dataset, the T5 model outperforms MQAN, suggesting a transformer-based language model is better suited to the decaNLP task. Additionally, the base model is more robust to paraphrasing, only loosing 42.1 of its total score when compared to the 140.1 lost by MQAN.

We find that MQAN trained on PQ-decaNLP (PQ-decaNLP trained) reduce this drop across all tasks except MWSC. We hypothesise that the lack of improvement in MWSC is because the original question: “*{mainQuestion} {choice1}* ***or*** *{choice2}*” already varies greatly between examples in the dataset-only the word ‘or’ separating the two choices is constant. For WikiSQL, we also find an improvement of 6.9 lf EM on the original dataset, suggesting that this task benefits from more varied questions.

When adding paraphrase detection or generation as an additional task, we find the MQAN model is able to learn these new tasks with 85.7 f1, and 31.4 bleu respectively. We find that while the tasks have little impact on the performance of the original decaNLP data (some scores are slightly higher), they perform worse than the original MQAN on PQ-decaNLP. This suggests that the knowledge learnt about paraphrasing does not help the robustness to paraphrasing of MQAN. Adding these new tasks significantly harms the performance of the T5 model on the original decaNLP data.

To better understand how the models behave on paraphrased questions, we conduct a range of analysis. We find only a weak negative correlation between the edit distance of the paraphrase (compared with the original question) and the score (R = −0.2373 for MQAN). This suggests that while many paraphrases which deviate further from the original question perform worse, other factors such as the type of paraphrase may also be significant.

### Impact of paraphrase phenomena

Table 4 shows the average difference in performance for paraphrases where a paraphrase phenomenon is present compared to those where the phenomenon is not present.

**Table 4:** Average difference in performance when paraphrase phenomena are present. Where the value is negative, paraphrases which contain this phenomena perform worse than those without. Entries marked with ‘-’ indicate where the task does not contain any examples of that type. We present the results X/Y where X is the performance difference on the original model, and Y is the performance difference of the PQ-decaNLP trained model.

Paraphrase type	MULTINLI	SST	IWSLT	WOZ	MWSC	CNN/DM	WIKISQL
**MQAN**							
Addition/deletion	−13.8/0.1	0.3/0.2	−1.0/0.0	−5.6/3.7	−0.3/1.5	−0.4/1.6	5.4/0.0
Same-polarity substitutions	17.8/−0.2	−5.5/−0.3	−1.9/0.0	−0.6/−5.7	0.6/−5.7	2.2/1.1	3.9/0.0
Sentence modality changes	2.3/ 0.0	5.3/−0.3	**−4.1**/−0.1	−1.1/0.7	1.6/−1.4	1.9/−0.8	**−7.6**/0.1
Synthetic/analytic substitutions	−0.2/−0.1	−5.3/0.3	0.1/0.0	−8.5/4.7	−3.5/−7.7	−0.1/1.0	4.5/0.0
Order	**−38.7**/−0.1	**−29.2**/−0.3	−3.2/−0.1	10.1/4.2	2.4/4.5	–	5.1/0.1
Punctuation changes	10.7/0.1	−15.2/−1.4	–	–	−5.0/−0.7	–	–
Semantics-based changes	–	−11.8/−0.1	−2.8/0.0	−3.6/3.9	–	−3.9/1.0	4.4/−0.1
Inflectional changes	–	−9.9/−0.5	–	−0.1/−6.1	–	0.0/0.9	–
Spelling changes	–	–	–	9.9/4.4	–	−2.5/−2.6	5.2/0.0
Ellipsis	−5.9/0.2	−14.5/−0.6	–	–	–	–	–
Coordination changes	19.0/0.1	–	–	–	−7.4/−3.3	–	–
Syntax/discourse structure changes	–	56.0/0.4	−2.9/0.0	−3.9/0.2	–	–	–
Modal verb changes	–	2.9/−0.1	–	–	–	–	–
**T5**							
Addition/deletion	1.3/1.4	0.0/0.2	−1.2/0.0	−3.9/−0.3	0.0/−1.2	−0.1/0.0	−0.1/0.0
Same-polarity substitutions	2.4/−1.5	0.0/−0.2	3.2/0.0	−3.2/−0.2	−4.2/−0.3	0.0/−0.1	0.0/0.0
Sentence modality changes	−1.4/0.0	−0.7/0.2	**−2.7**/−0.0	−6.3/−0.6	−4.2/4.7	−0.4/−0.1	**0.0**/0.0
Synthetic/analytic substitutions	−0.1/0.2	−0.2/0.2	−3.3/−0.1	2.8/0.7	2.2/0.7	−0.1/0.1	−0.1/0.0
Order	**0.3**/0.2	**−0.3**/−0.1	0.7/−0.1	1.7/0.1	−4.2/−6.6	–	−0.1/−0.1
Punctuation changes	−1.6/0.0	0.5/0.3	–	–	−3.6/−5.6	–	–
Semantics-based changes	–	−0.7/−0.2	−9.4/0.0	8.8/0.0	–	0.1/0.0	0.2/0.0
Inflectional changes	–	−0.2/0.2	–	−3.7/−0.8	–	0.2/0.0	–
Spelling changes	–	–	–	9.7/0.7	–	0.2/−0.1	0.3/0.0
Ellipsis	3.2/1.1	−0.1/0.2	–	–	–	–	–
Coordination changes	−12.3/−3.6	–	–	–	−7.5/−10.1	–	–
Syntax/discourse structure changes	–	0.6/0.0	1.2/0.0	−6.1/2.5	–	–	–
Modal verb changes	–	−0.8/−0.2	–	–	–	–	–

Paraphrased questions which contain an ‘Order’ annotation perform worse in classification tasks with fixed labels (MNLI, SST) than other paraphrases when using MQAN. The ‘Order’ tag occurs in 73% of the sentiment analysis task (SST), primarily in the swapping of the class labels ‘positive’ and ‘negative’. We find that manually swapping the class labels back to the same order as the original question, increased the performance by 38.2 (to 61.6), suggesting the model is memorising the position of the labels rather than their semantics. This sensitivity to label order harms the ability of MQAN to perform zero-shot learning. The T5 model looses less performance on MNLI and SST, and we no longer find that ‘Order’ paraphrases are causing the largest decrease in performance, suggesting the trained T5 model is much more robust to label position and is comprehending their meaning.

An interesting aspect of the original decaNLP framing is that English to SQL translation is formed as a question (“What is the translation from English to SQL?”), where English to German translation is framed as an imperative command (“Translate from English to German.”). We see from Table 4 that MQAN performs especially poorly on WikiSQL and IWSLT paraphrases which contain a change in sentence modality. Inspecting the answers for these WikiSQL cases reveals that the model outputs German words, indicating confusion between English-SQL translation and English-German translation. This suggests the model overly relies on the indicators of sentence modality (“What is the”, “translate”/“translation”) rather than the source and target languages. Again we see that the T5 model is more resilient to changes of sentence modality.

We find that the models trained on PQ-decaNLP have a smaller range of performance, suggesting they perform similarly across all paraphrase types.

We find no correlation between the number of phenomena present and the score.

### Analysing pointers

**Figure 6:**
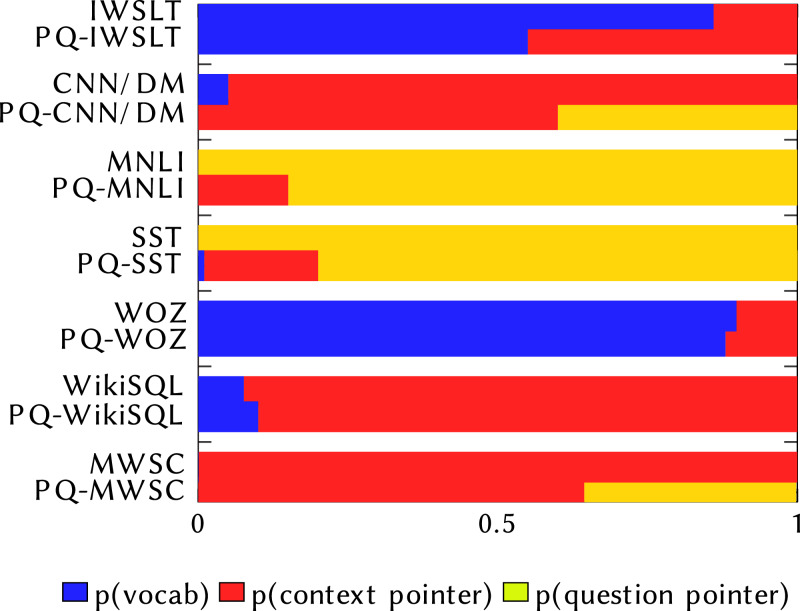
Comparison of where MQAN selects output from. The top bar for each dataset shows the pointer values on the original decaNLP dataset, while the bottom one shows the values when evaluated on PQ-decaNLP.

To gain a better insight into why MQAN fails, we analyse where the model copies its answers from: the question, the context, or the external vocabulary, which we present in Fig. 6. By analysing the values of the model’s learned pointers, we can better understand how it makes errors-Does the model pick words from the wrong source indicating that it is confused about what kind of task it is being asked to solve, or simply pick an incorrect word when solving the correct task? We confirm the findings of McCann et al. (2018) that the MQAN does not confuse tasks when asked the original decaNLP questions. However, when evaluated on the paraphrased questions we see more confusion. For the classification tasks (MNLI, SST, MWSC), where the class labels are contained within the question, we see a decrease in copying from the question. For translation (IWSLT), we see an increase in copying from the context, and for semantic parsing (WikiSQL) we see an increase in copying from the external vocabulary. These indicate the confusion between these tasks.

## Conclusion

In this work we explore how robust text-to-text models are to paraphrasing of questions asked. We introduce a diagnostic *corpus* annotated with paraphrase phenomena and show how simple transformations such as changing the label order and altering the sentence modality can harm the performance. We believe that the creation of similar typology-annotated corpora will provide useful insights into the robustness to paraphrasing of many models across NLP.

Additionally, we find that training models on paraphrased questions improves its robustness to paraphrasing. We find that knowledge learnt from adding the tasks of paraphrase generation or paraphrase detection does not transfer to increased robustness in other tasks for either model.

We hope that the paraphrase *corpus* of decaNLP questions will encourage further research into more robust multitask question answering models.

## Additional Information and Declarations

### Data Availability

The following information was supplied regarding data availability: The raw data is available at GitHub: https://github.com/ghomasHudson/paraphraseDecanlpCorpus.
